# Supplementary material for: Explainable AI: Machine Learning Interpretation in Blackcurrant Powders
Source: Sensors (Basel). 2024 May 17;24(10):3198. doi: 10.3390/s24103198 (PMC11124776; doi:10.3390/s24103198)
Supplement: Supplementary file 1 [file sensors-24-03198-s001.zip › sensors-2943799-supplementary.pdf]

sensors

MDPI

Article

Explainable AI: machine learning interpretation in blackcurrant powders

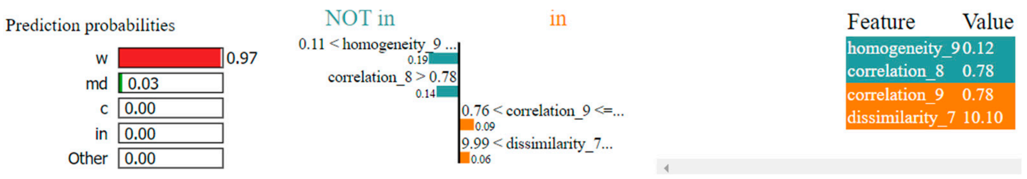

(a)

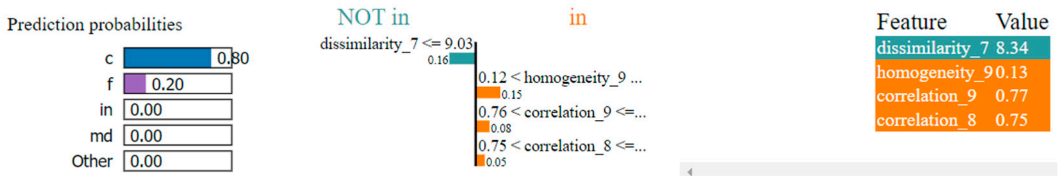

(b)

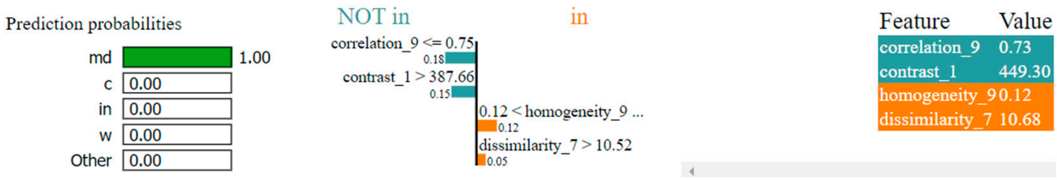

(c)

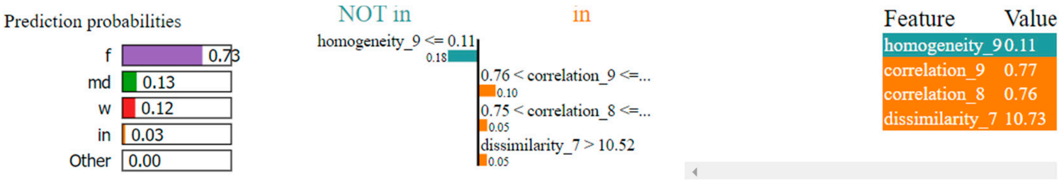

(d)

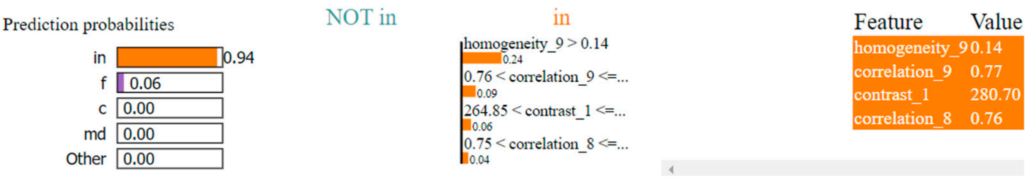

(e)

Figure S1. Explanation for the test sample with indexes 50 (a), 127 (b), 2 (c), 114 (d) and 178 (e) by DT5 model.

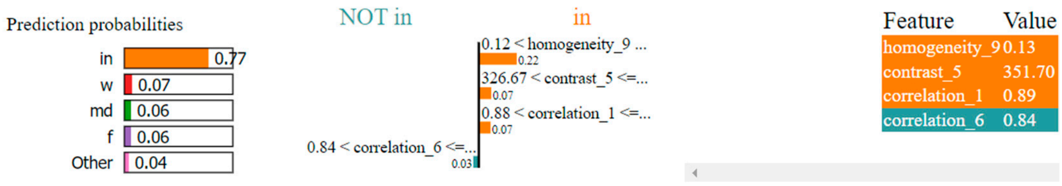

(a)

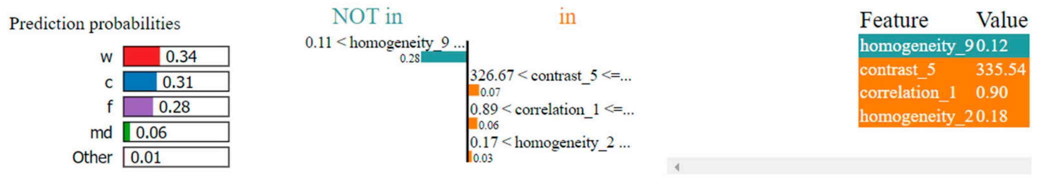

(b)

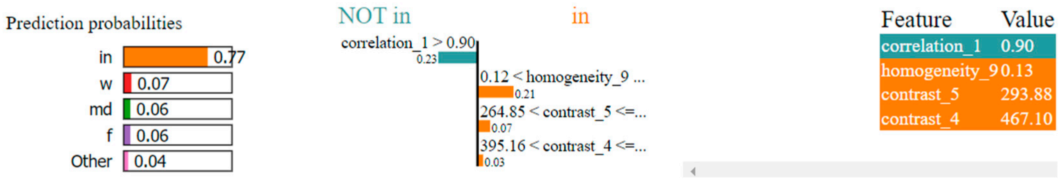

(c)

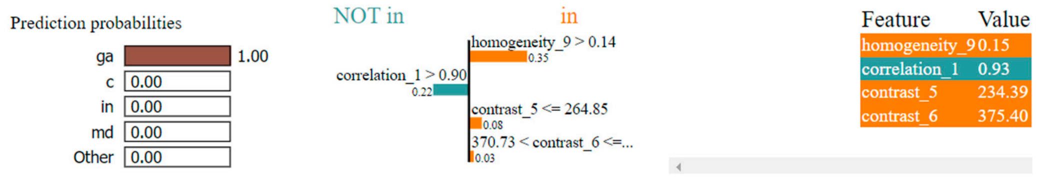

(d)

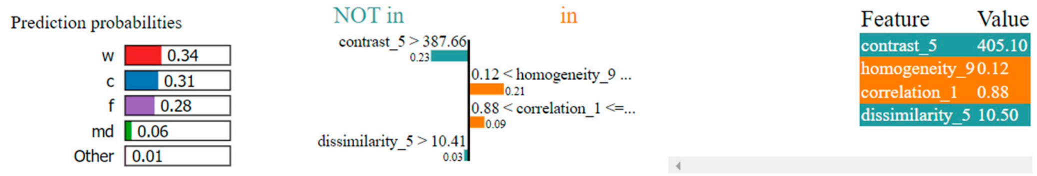

(e)

Figure S2. Explanation for the test sample with indexes 54 (a), 84 (b), 57 (c), 139(d) and 168 (e) by DT3 model.

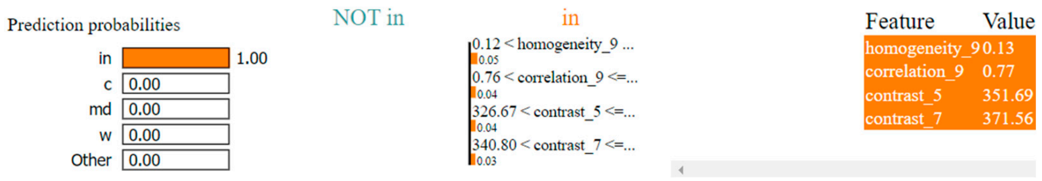

(a)

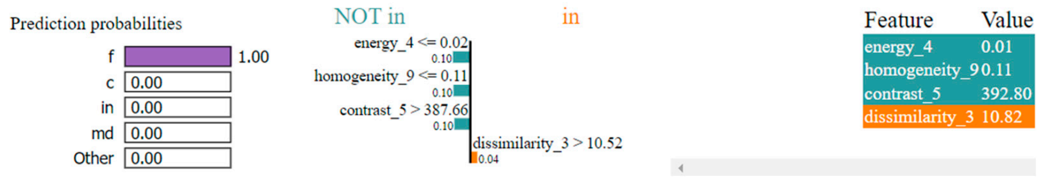

(b)

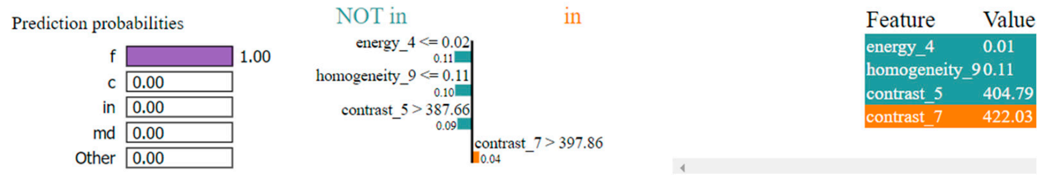

(c)

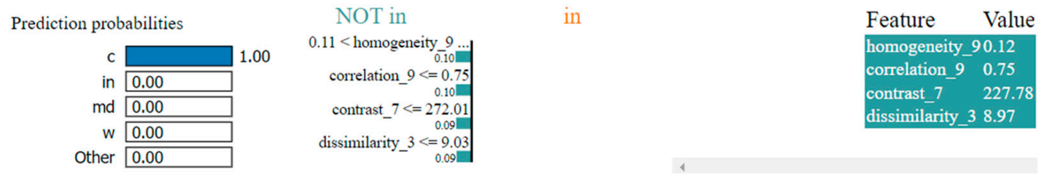

(d)

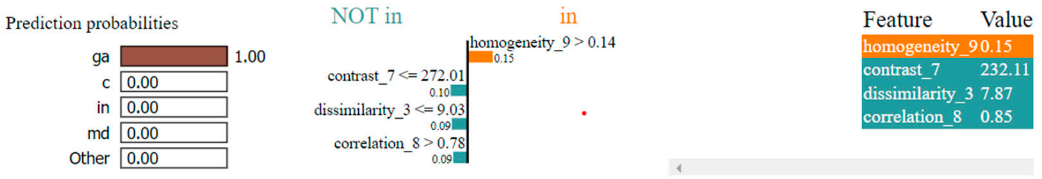

(e)

Figure S3. Explanation for the test sample with indexes 133 (a), 98 (b), 117 (c), 42 (d) and 44 (e) by DT_best model.

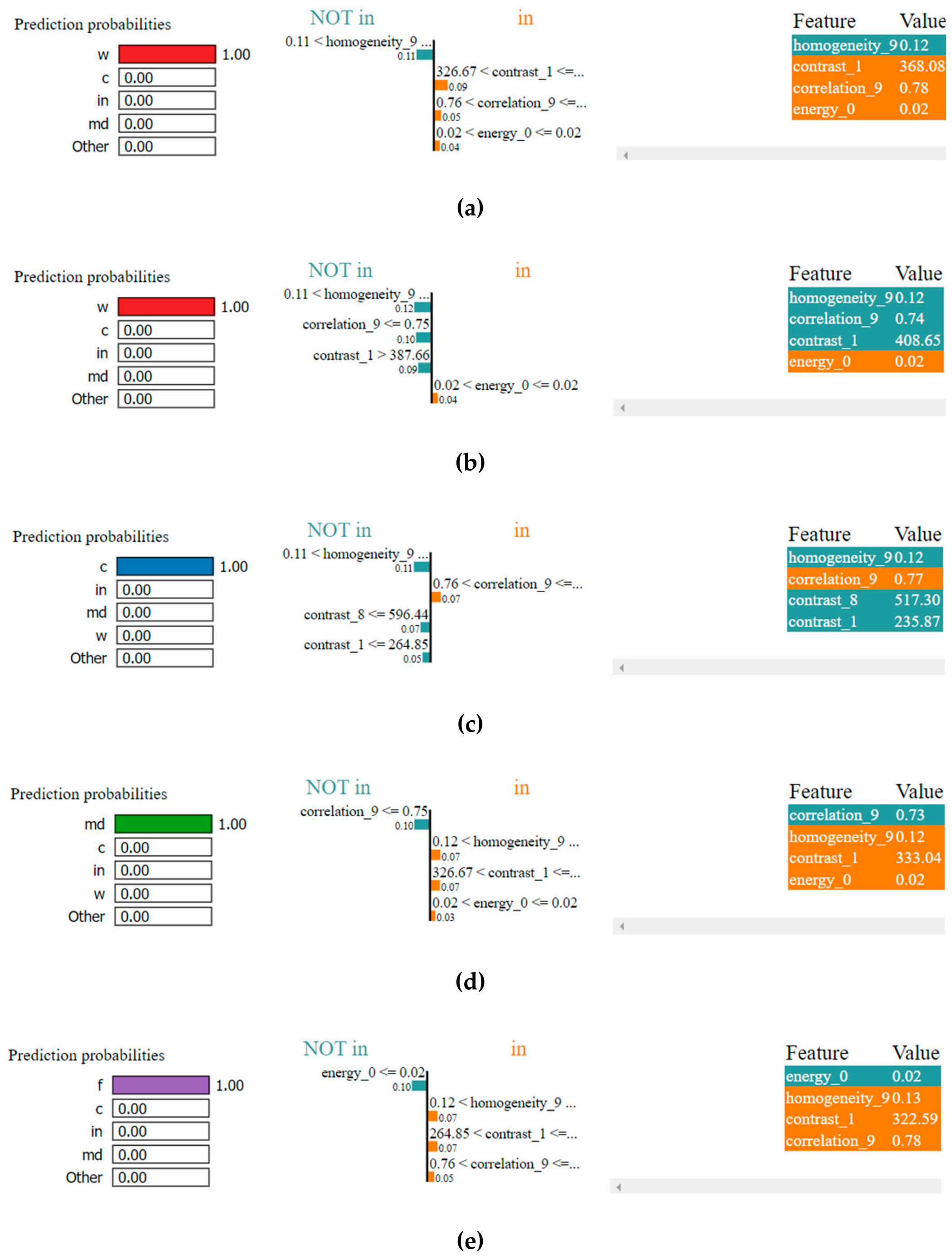

Figure S4. Explanation for the test sample with indexes 188 (a), 115 (b), 71 (c), 27 (d) and 58 (e) by DT0 model.

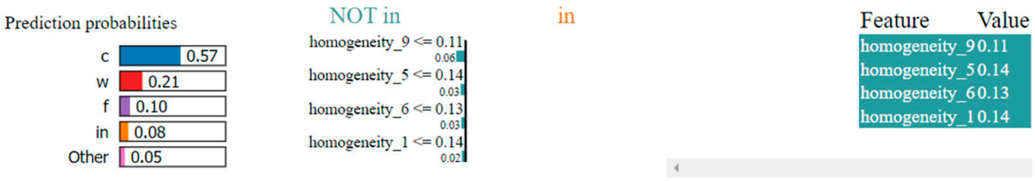

(a)

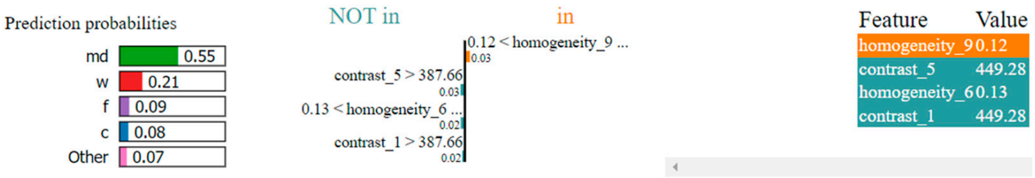

(b)

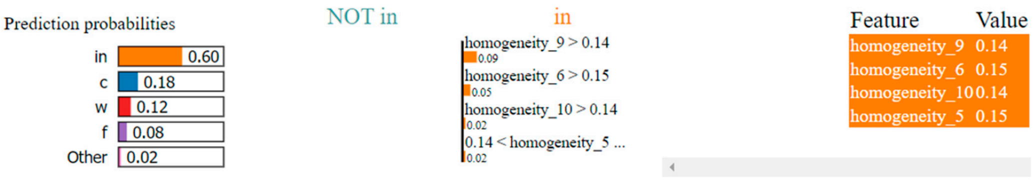

(c)

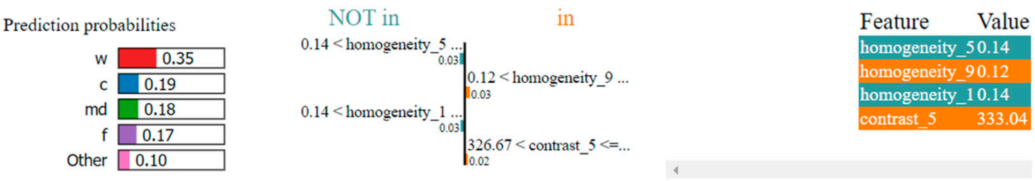

(d)

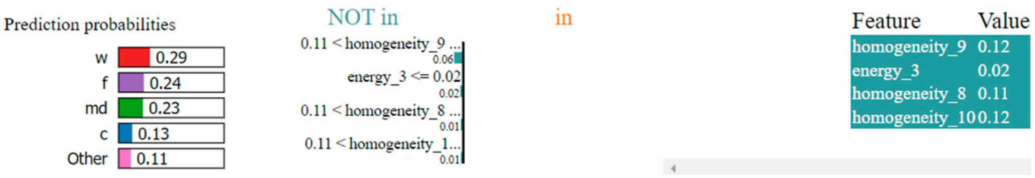

(e)

Figure S5. Explanation for the test sample with indexes 59 (a), 171(b), 91 (c), 27 (d) and 102 (e) by RF3_gini model.

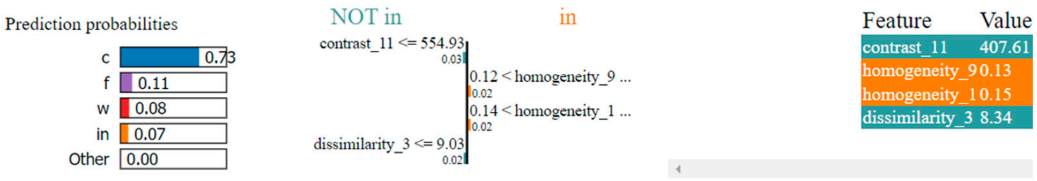

(a)

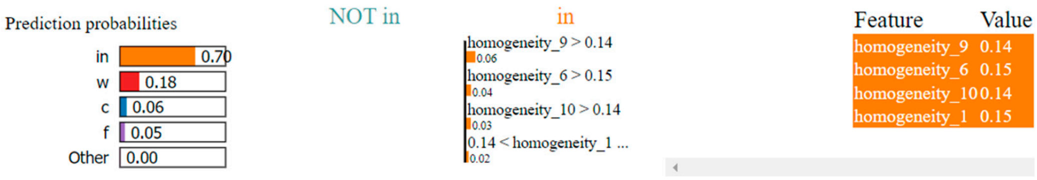

(b)

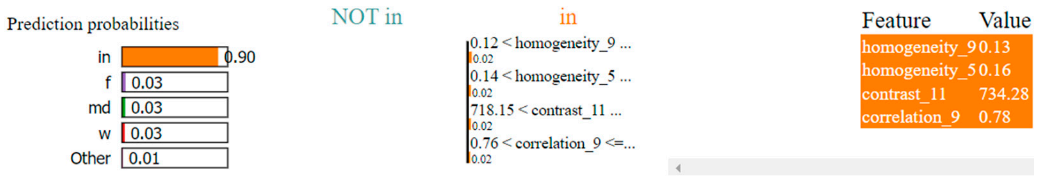

(c)

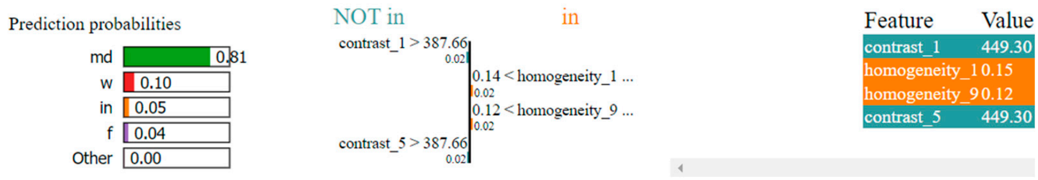

(d)

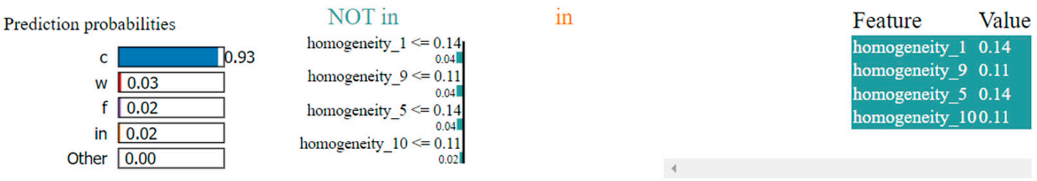

(e)

Figure S6. Explanation for the test sample with indexes 127 (a), 91 (b), 161 (c), 47 (d) and 94 (e) by RF5_gini model.

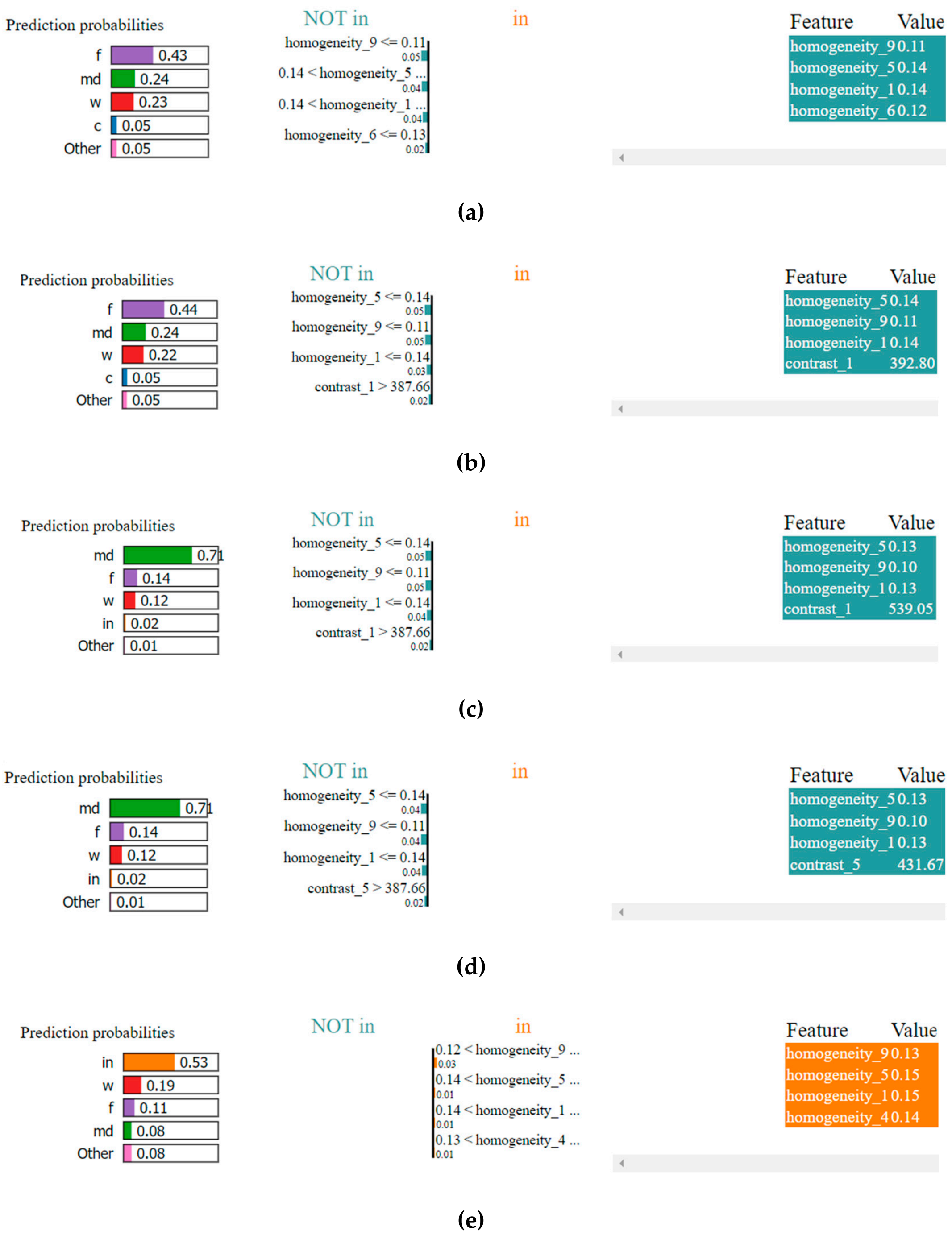

Figure S7. Explanation for the test sample with indexes 114 (a), 162 (b), 19 (c), 113 (d) and 61 (e) by RF3 model.

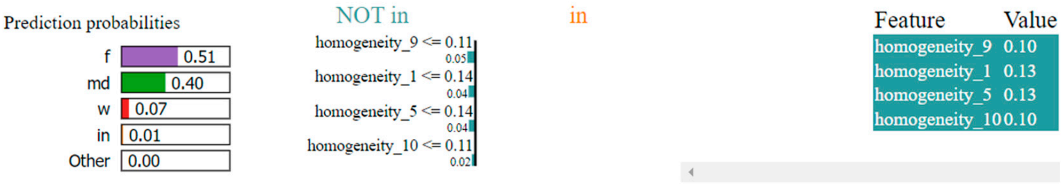

(a)

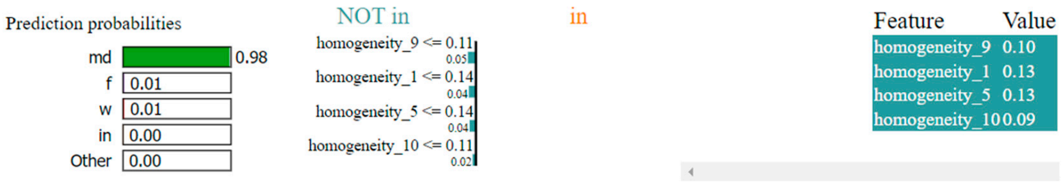

(b)

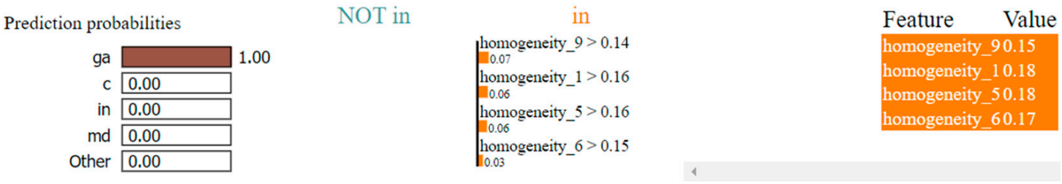

(c)

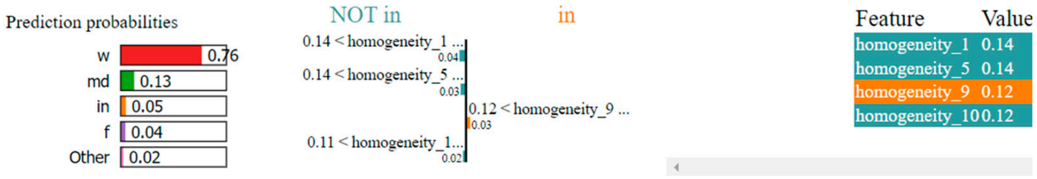

(d)

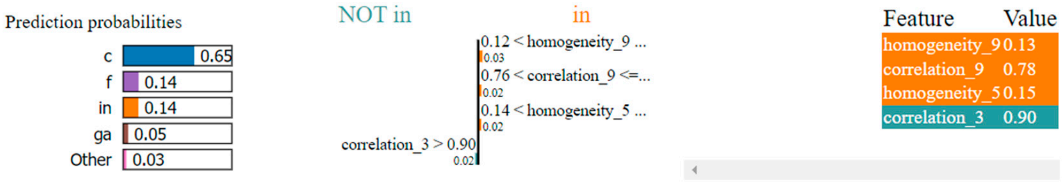

(e)

Figure S8. Explanation for the test sample with indexes 3 (a), 37 (b), 43 (c), 168 (d) and 112 (e) by RF5 model.

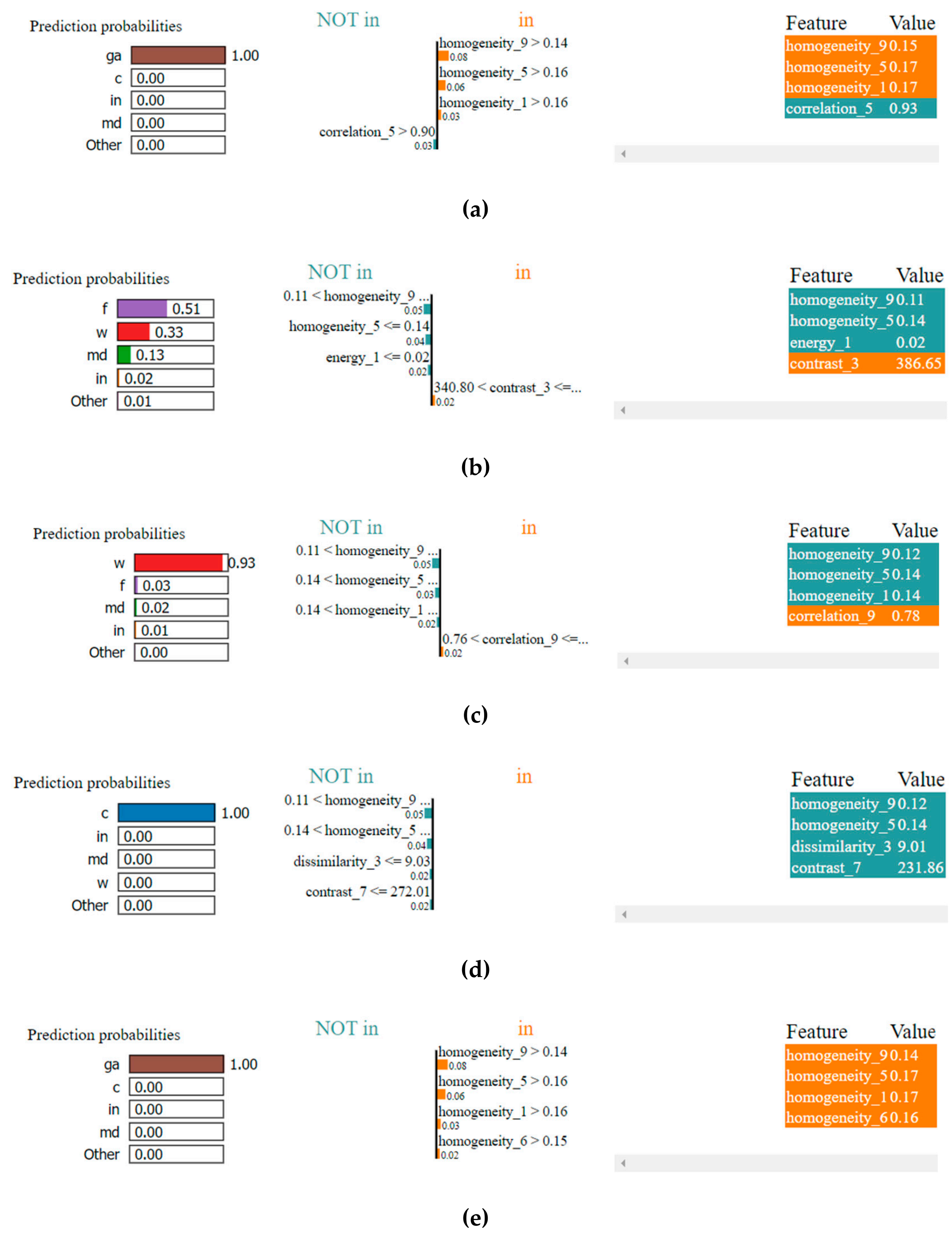

Figure S9. Explanation for the test sample with indexes 151 (a), 182 (b), 188 (c), 71 (d) and 175 (e) by RF7_gini model.

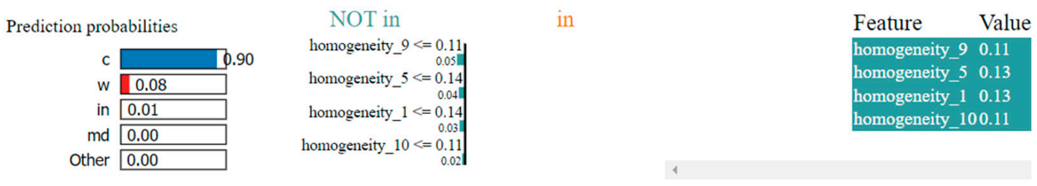

(a)

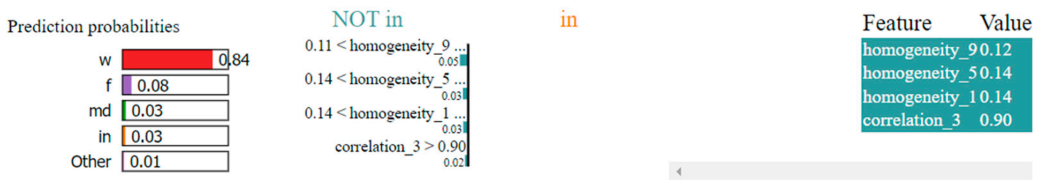

(b)

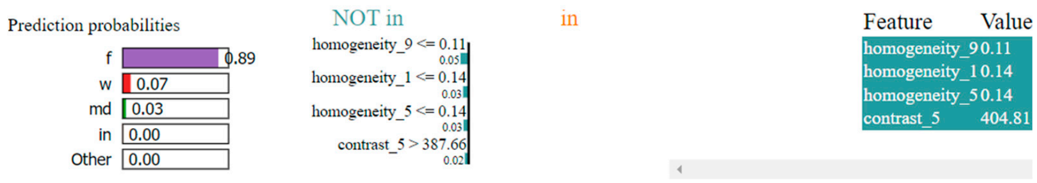

(c)

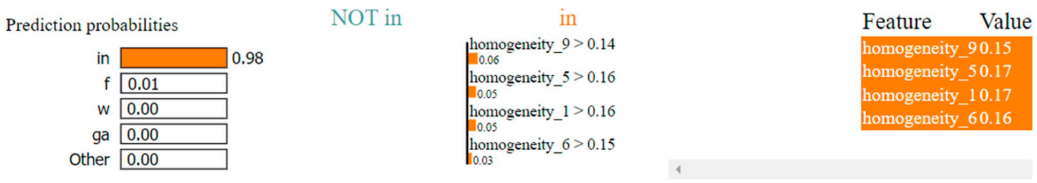

(d)

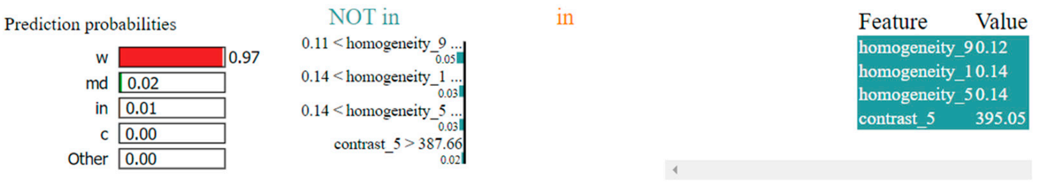

(e)

Figure S10. Explanation for the test sample with indexes 105 (a), 25 (b), 53 (c), 36 (d) and 55 (e) by RF7 model.

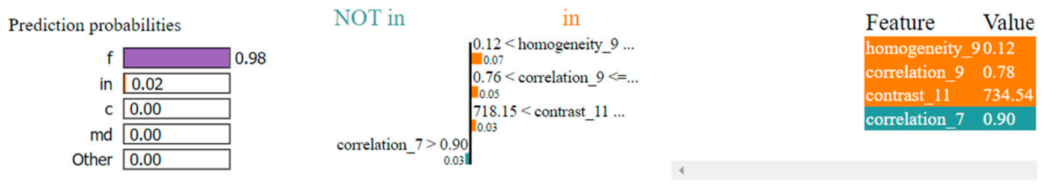

(a)

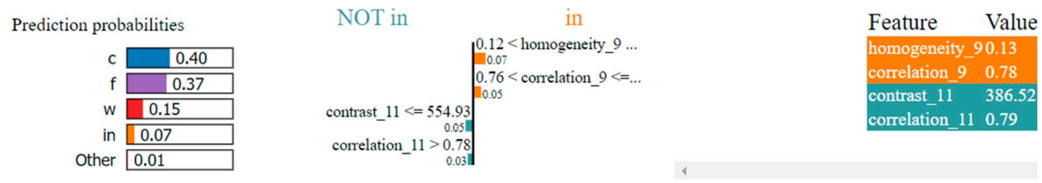

(b)

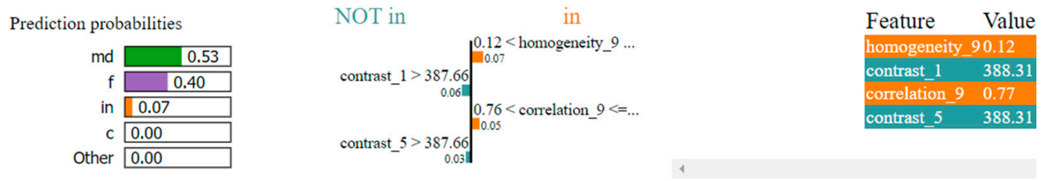

(c)

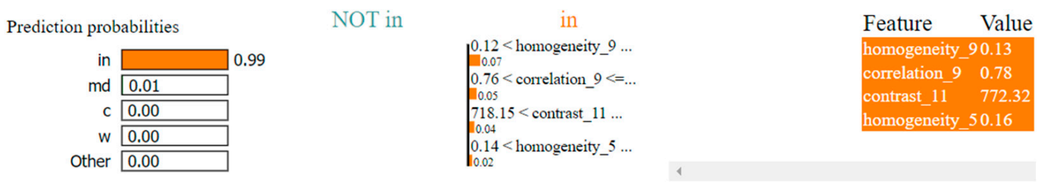

(d)

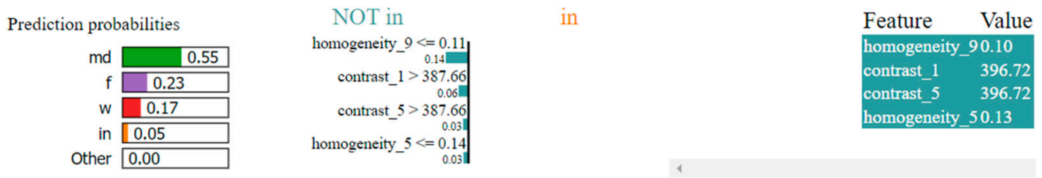

(e)

Figure S11. Explanation for the test sample with indexes 104 (a), 143 (b), 103 (c), 176 (d) and 116 (e) by Bagging_100 model.

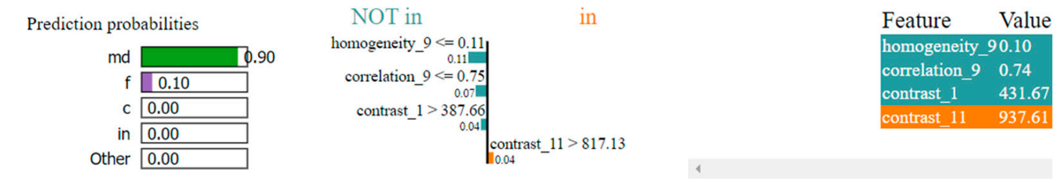

(a)

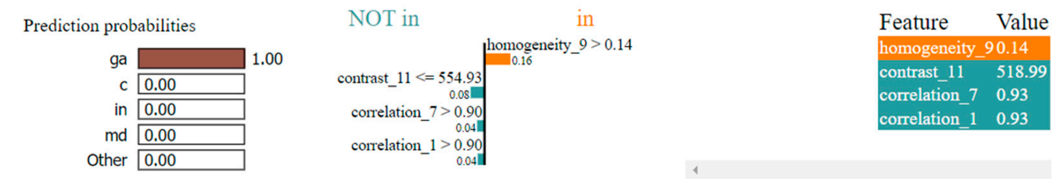

(b)

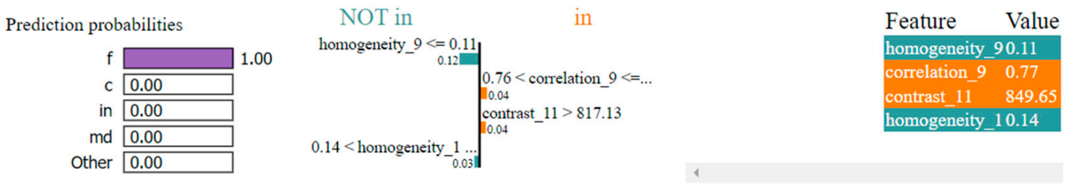

(c)

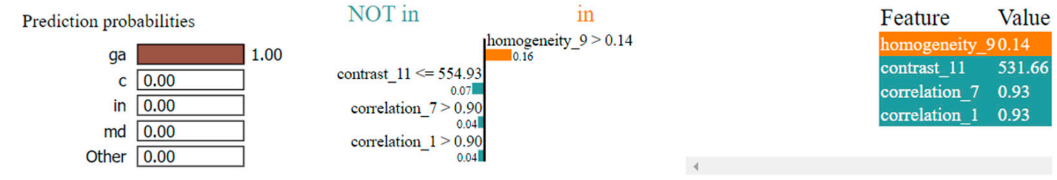

(d)

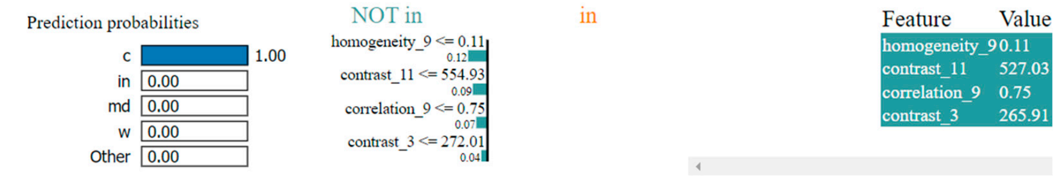

(e)

Figure S12. Explanation for the test sample with indexes 113 (a), 67 (b), 114 (c), 35 (d) and 81 (e) by Bagging model.

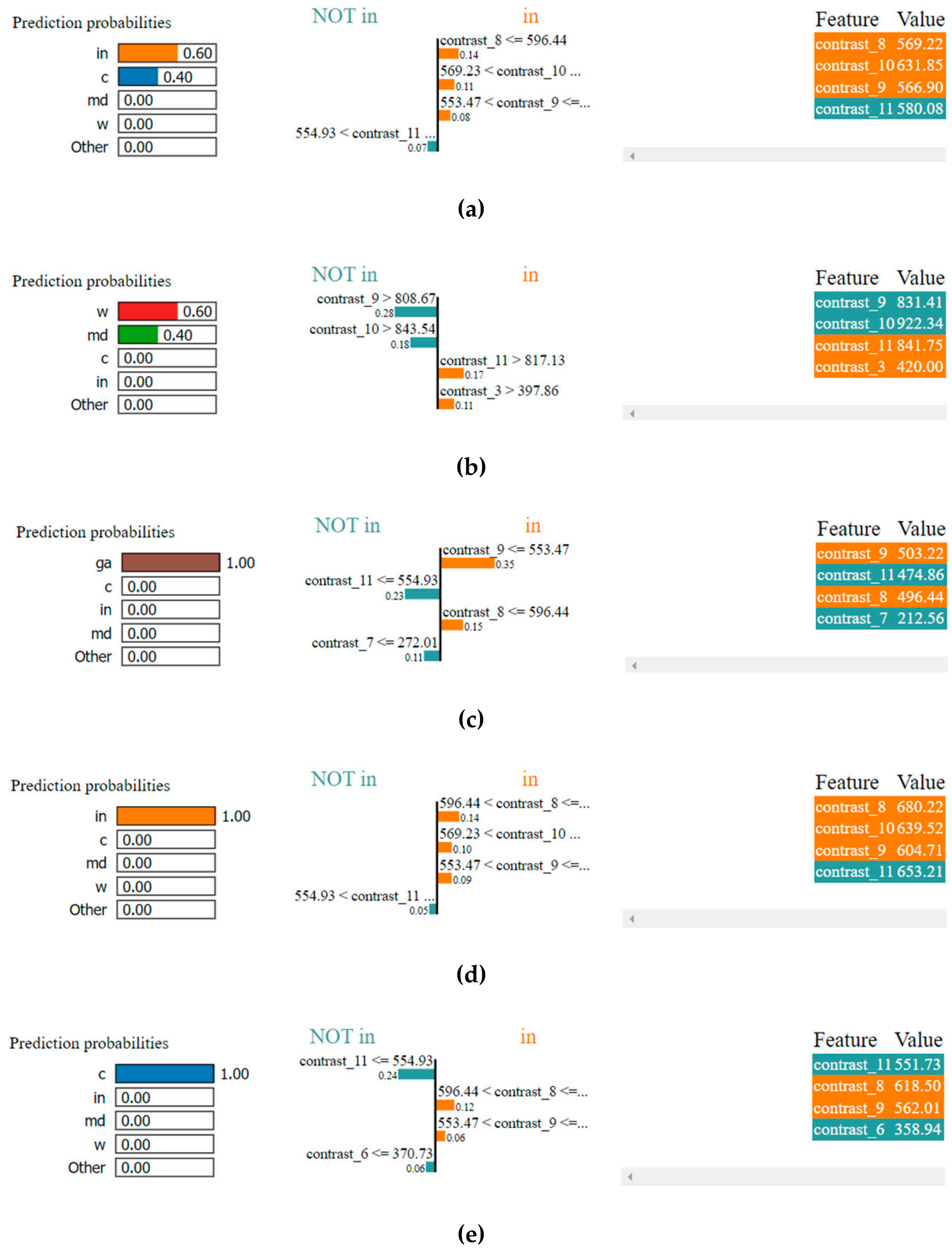

Figure S13. Explanation for the test sample with indexes 166 (a), 6 (b), 150 (c), 57 (d) and 186 (e) by KNN model.

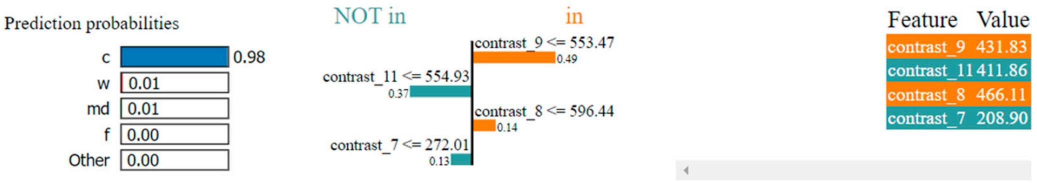

(a)

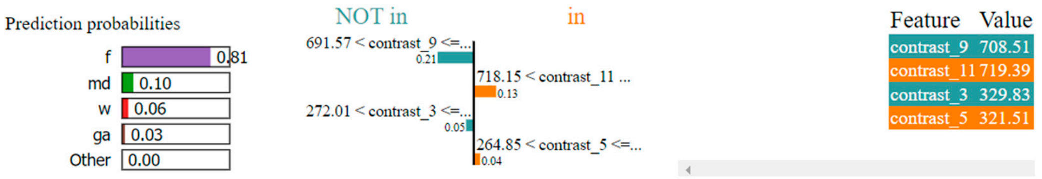

(b)

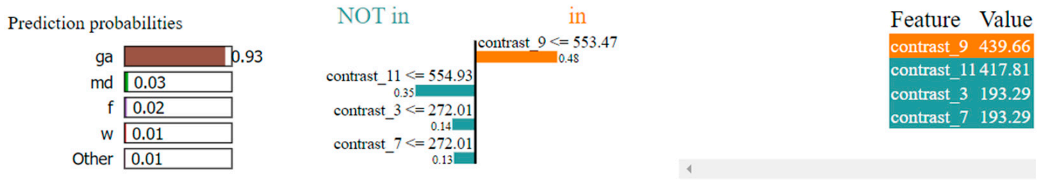

(c)

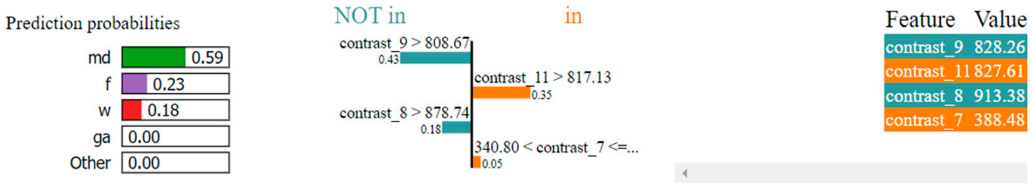

(d)

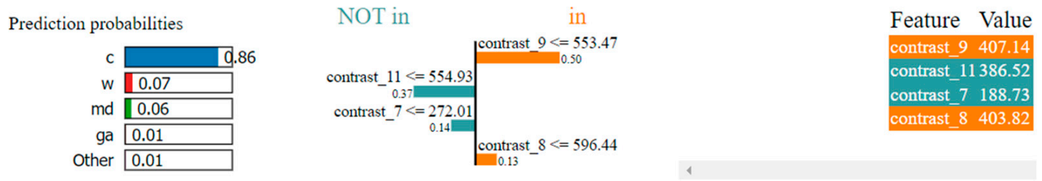

(e)

Figure S14. Explanation for the test sample with indexes 187 (a), 25 (b), 43 (c), 132 (d) and 143 (e) by LogReg model.
