# Supplementary figures and images for: Explainable AI: Machine Learning Interpretation in Blackcurrant Powders
Source: Sensors (Basel). 2024 May 17;24(10):3198. doi: 10.3390/s24103198 (PMC11124776; doi:10.3390/s24103198)

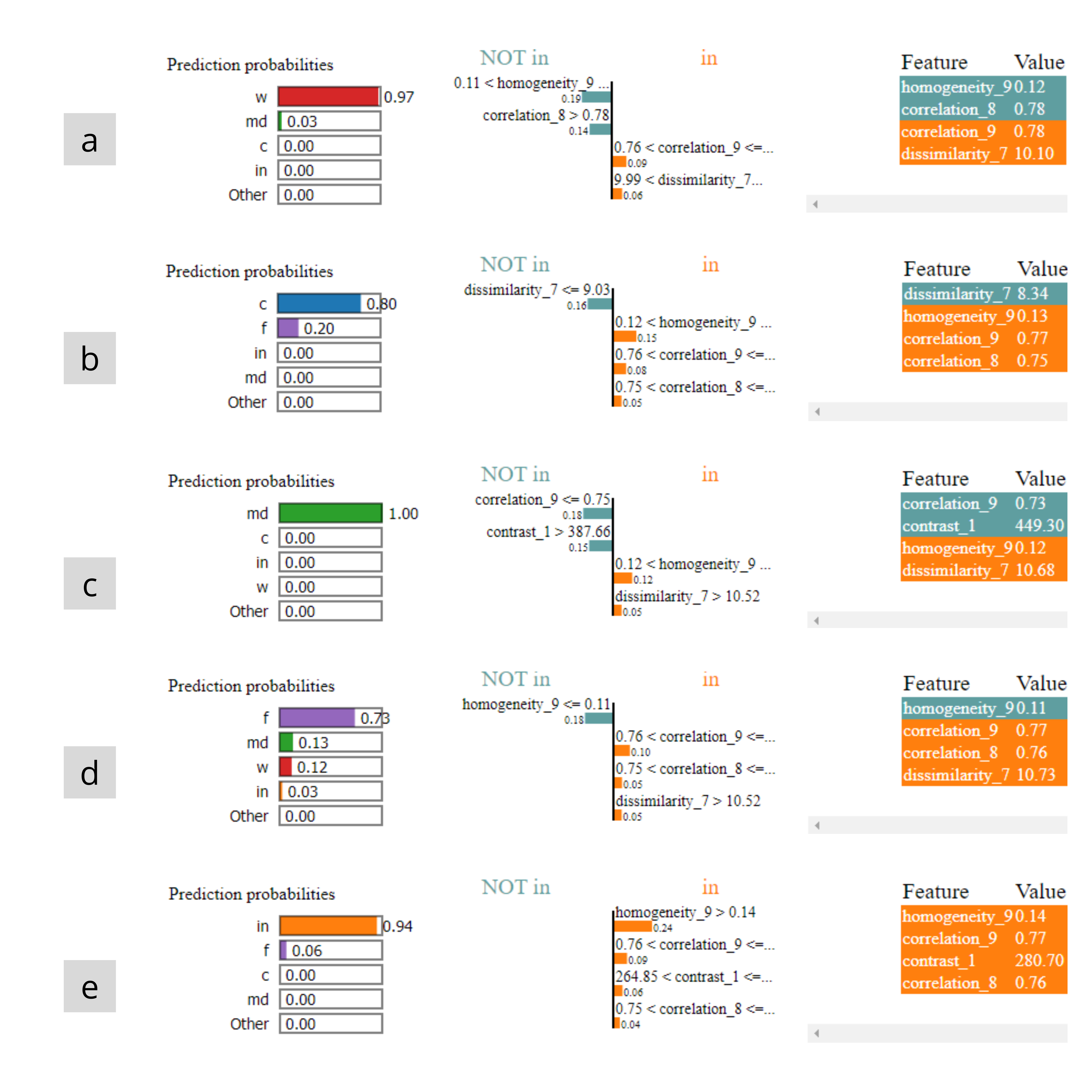

Supplement: Supplementary file 1 [file sensors-24-03198-s001.zip › FigureS1.png]

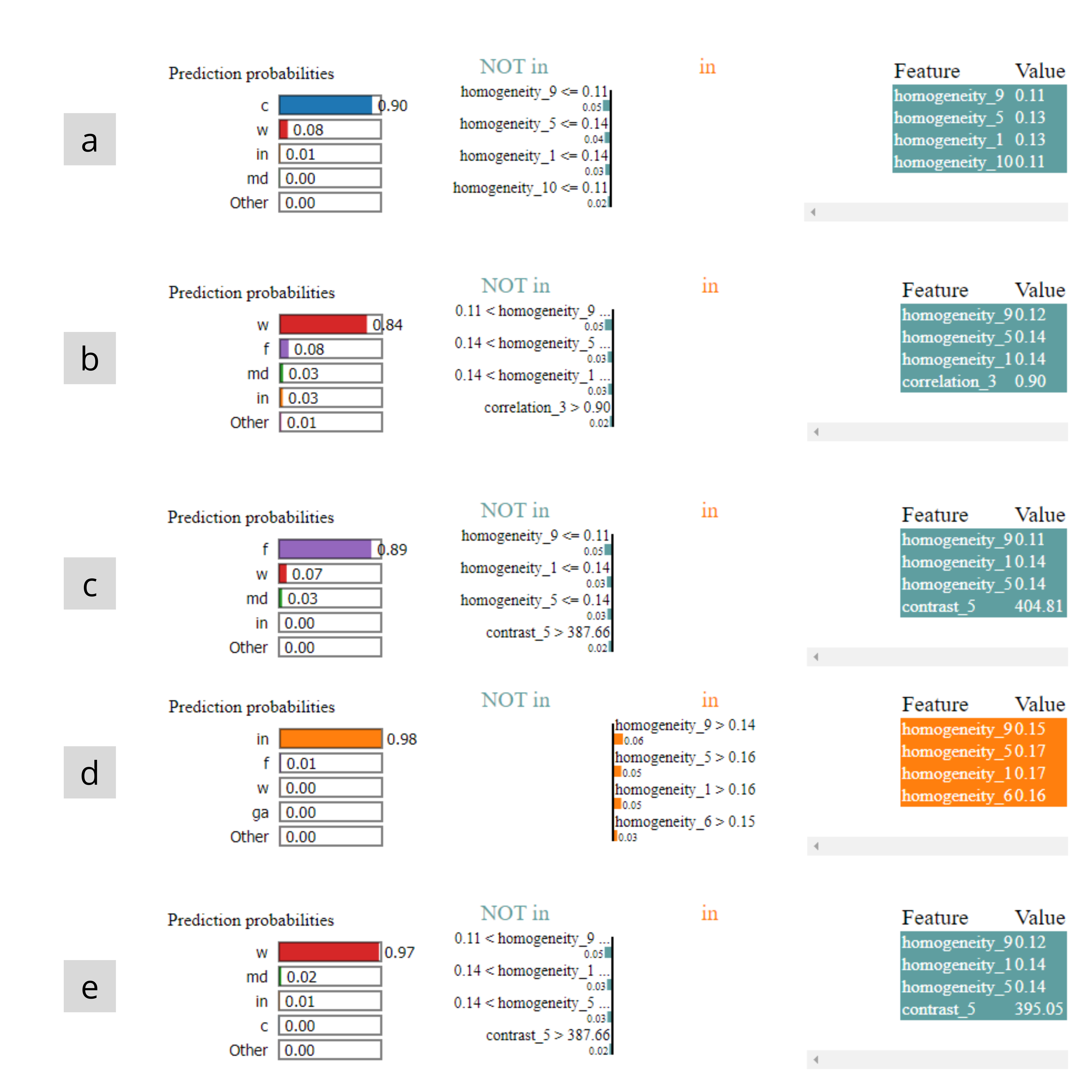

Supplement: Supplementary file 1 [file sensors-24-03198-s001.zip › FigureS10.png]

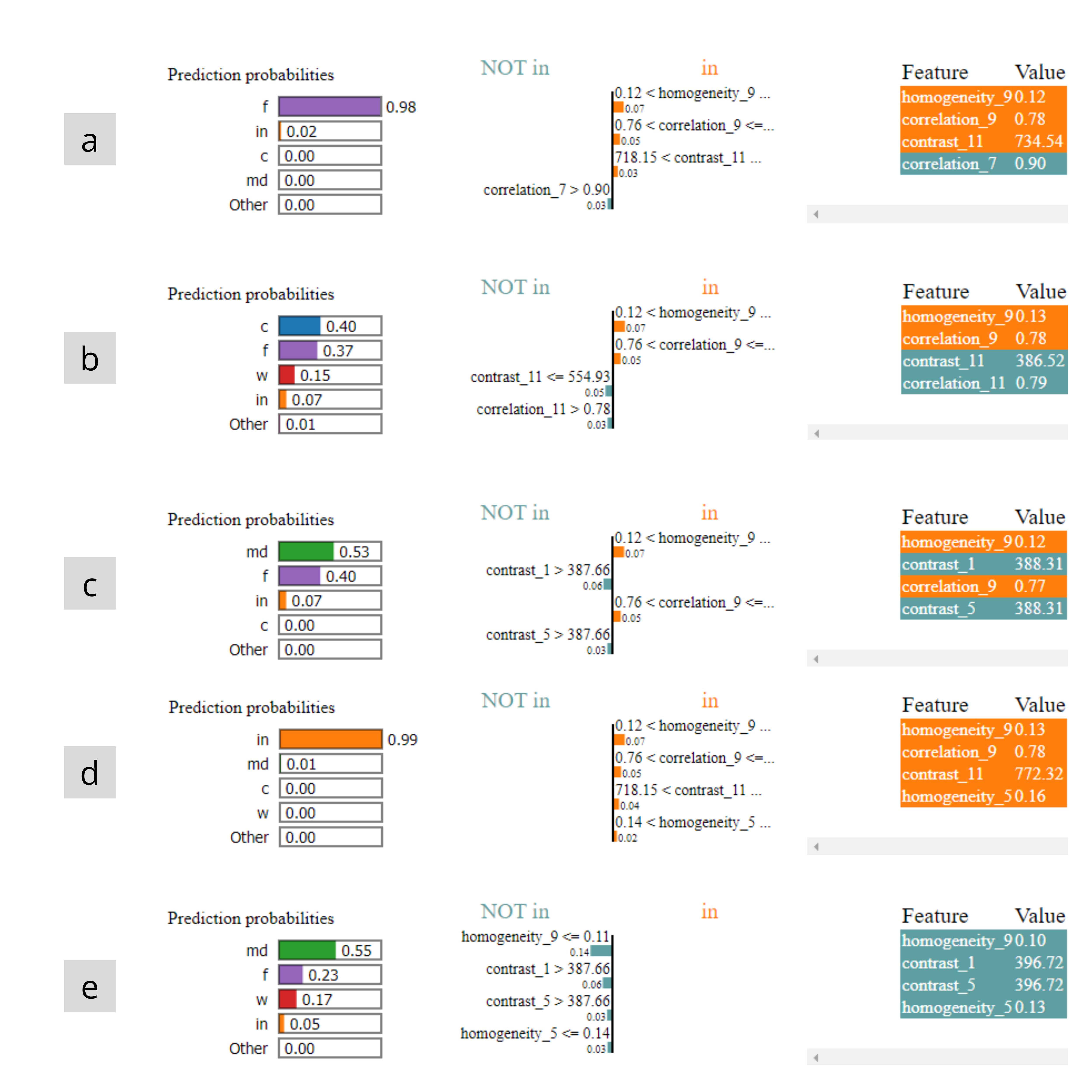

Supplement: Supplementary file 1 [file sensors-24-03198-s001.zip › FigureS11.png]

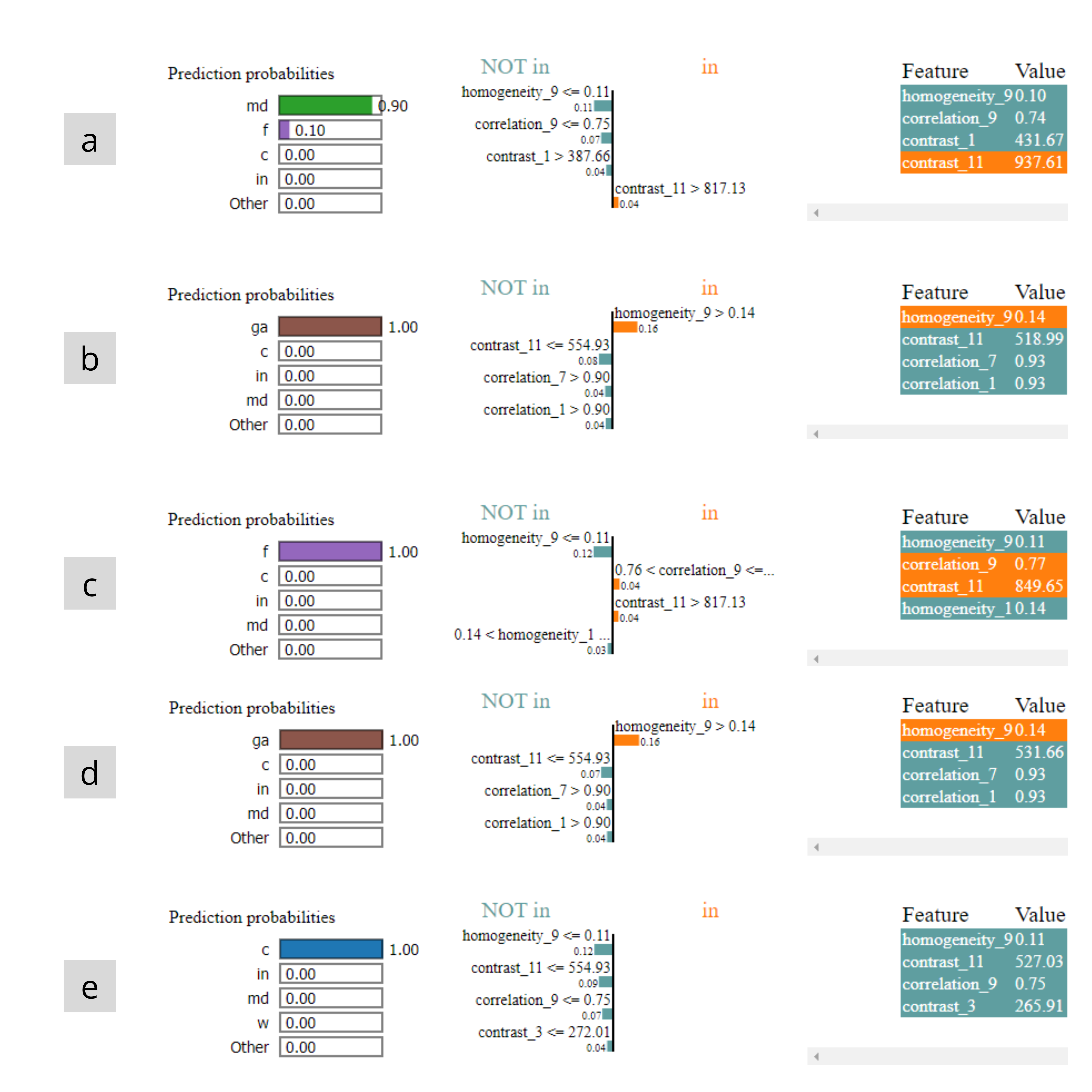

Supplement: Supplementary file 1 [file sensors-24-03198-s001.zip › FigureS12.png]

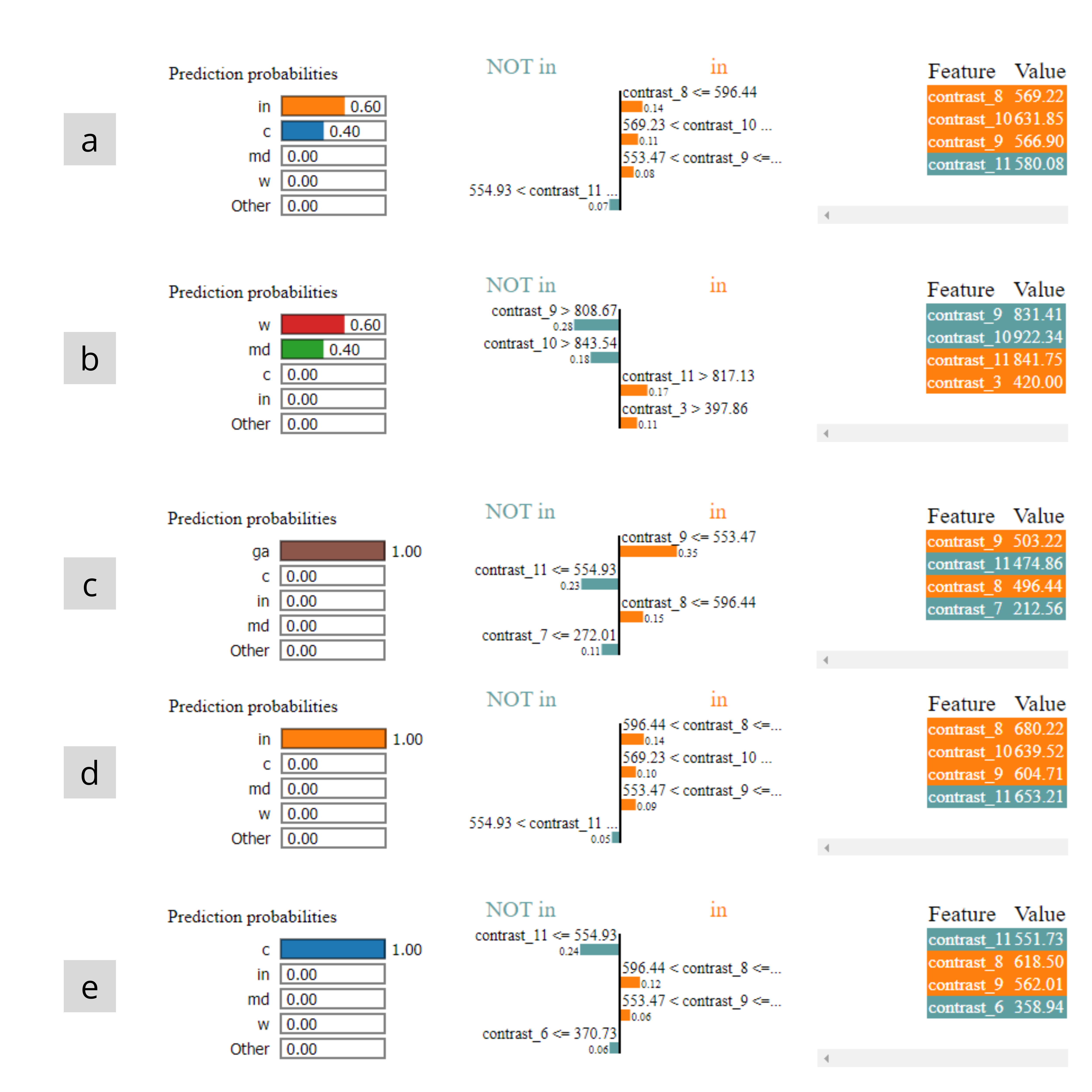

Supplement: Supplementary file 1 [file sensors-24-03198-s001.zip › FigureS13.png]

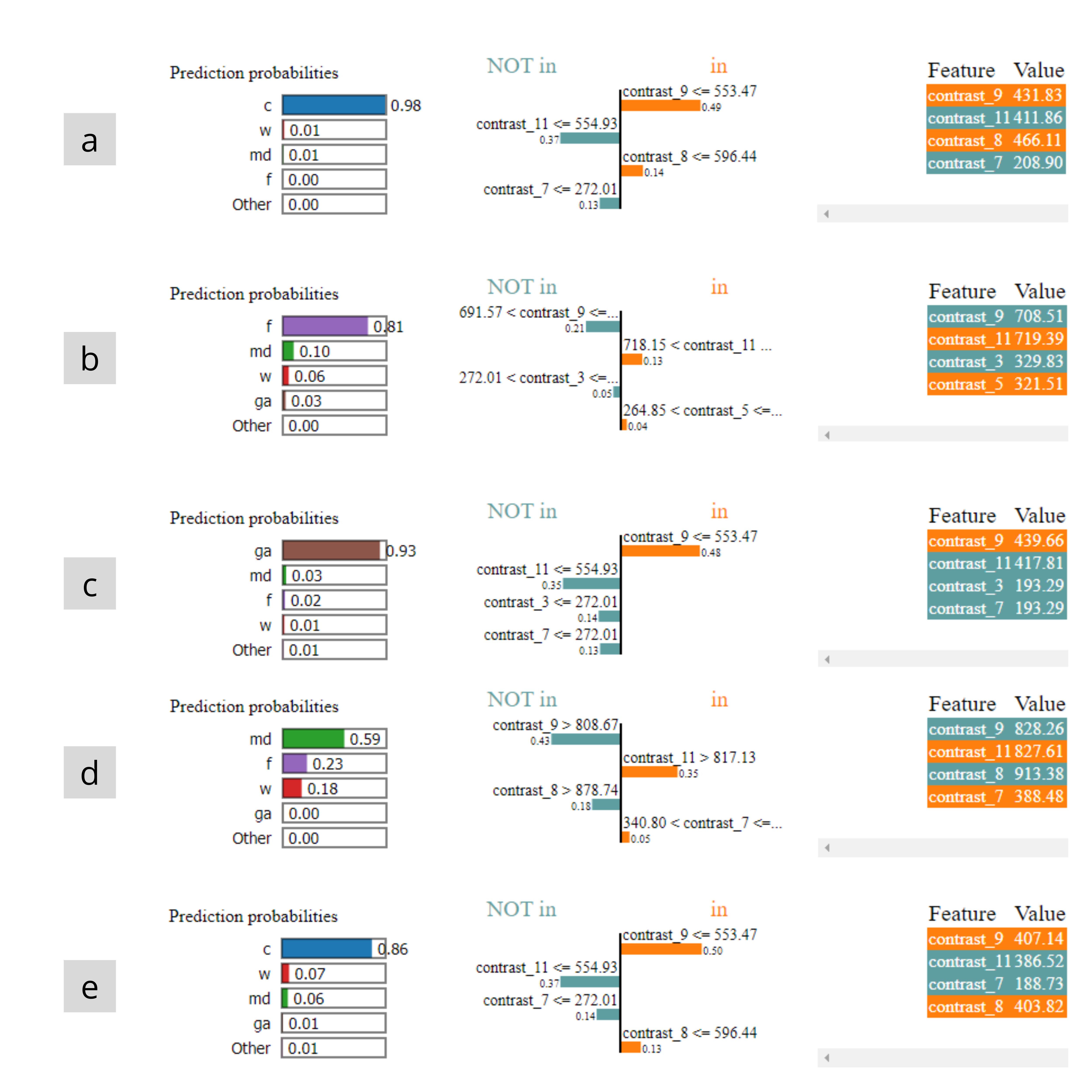

Supplement: Supplementary file 1 [file sensors-24-03198-s001.zip › FigureS14.png]

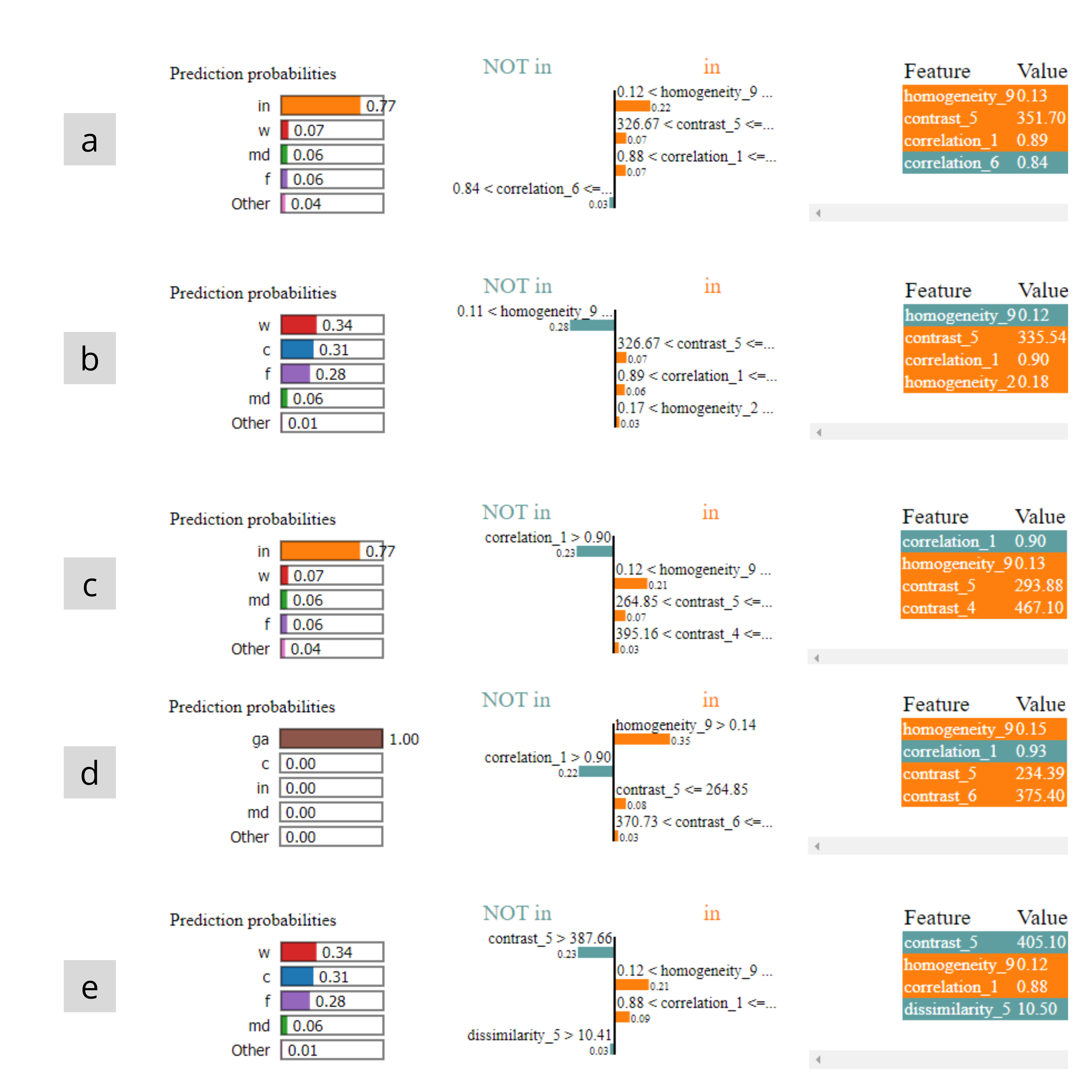

Supplement: Supplementary file 1 [file sensors-24-03198-s001.zip › FigureS2.png]

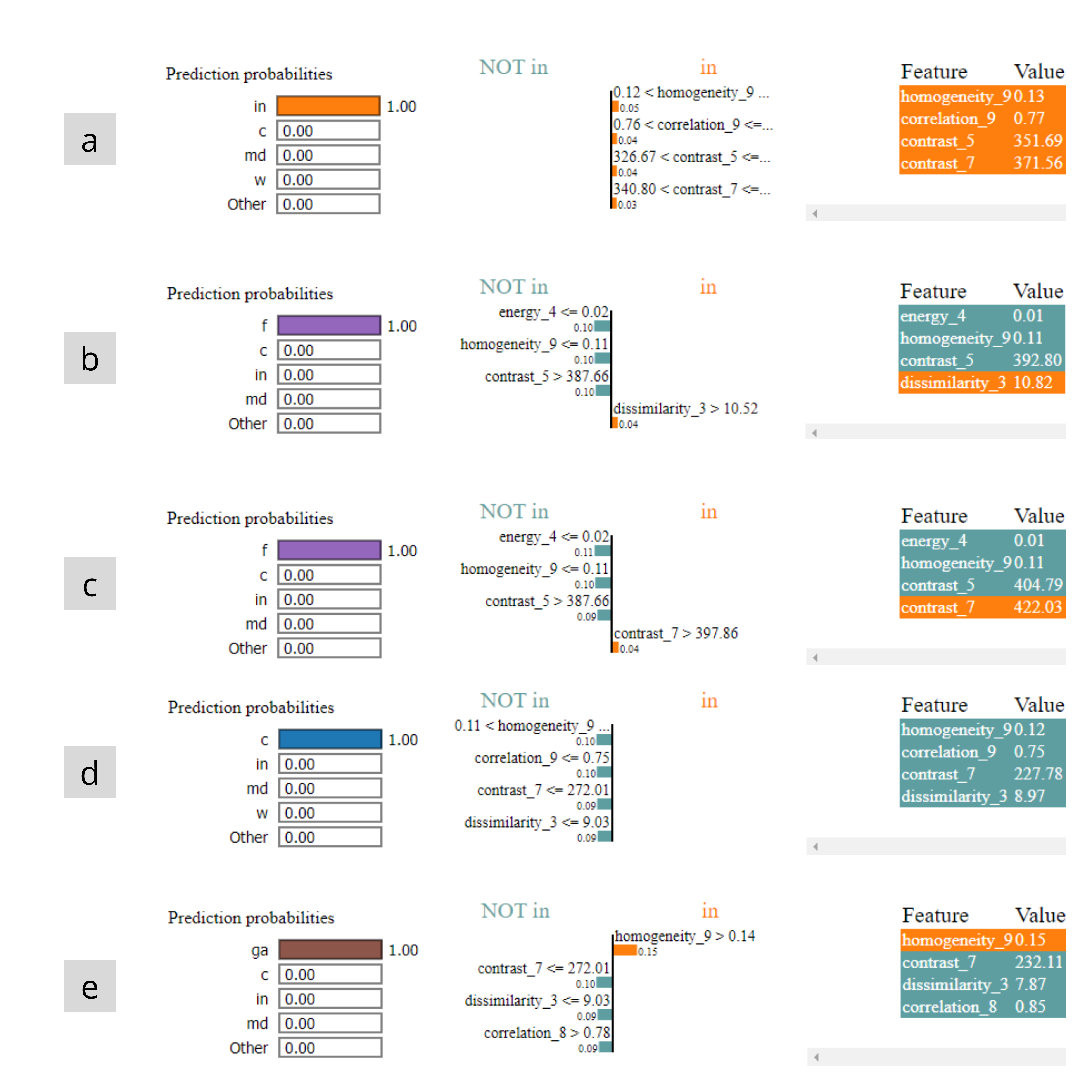

Supplement: Supplementary file 1 [file sensors-24-03198-s001.zip › FigureS3.png]

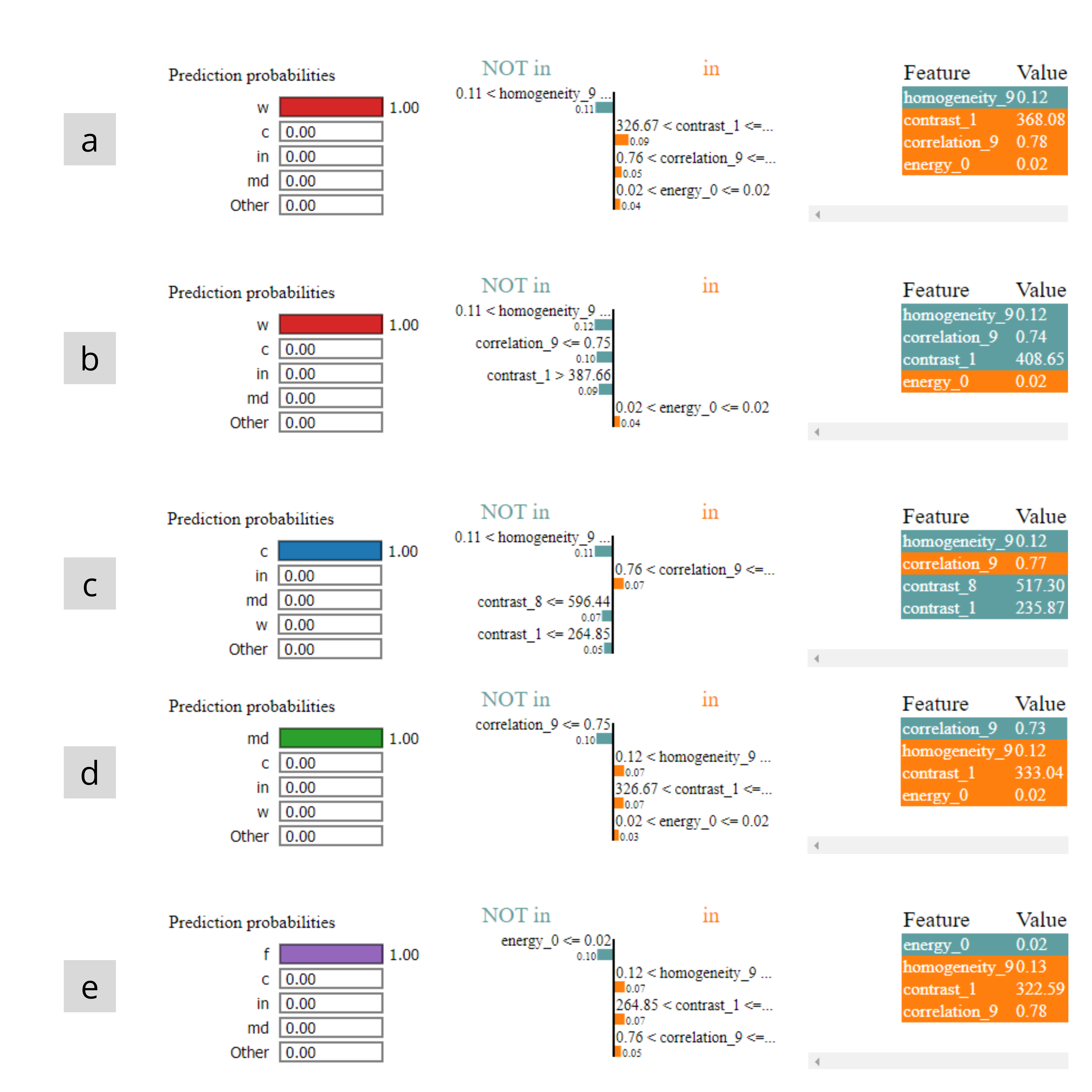

Supplement: Supplementary file 1 [file sensors-24-03198-s001.zip › FigureS4.png]

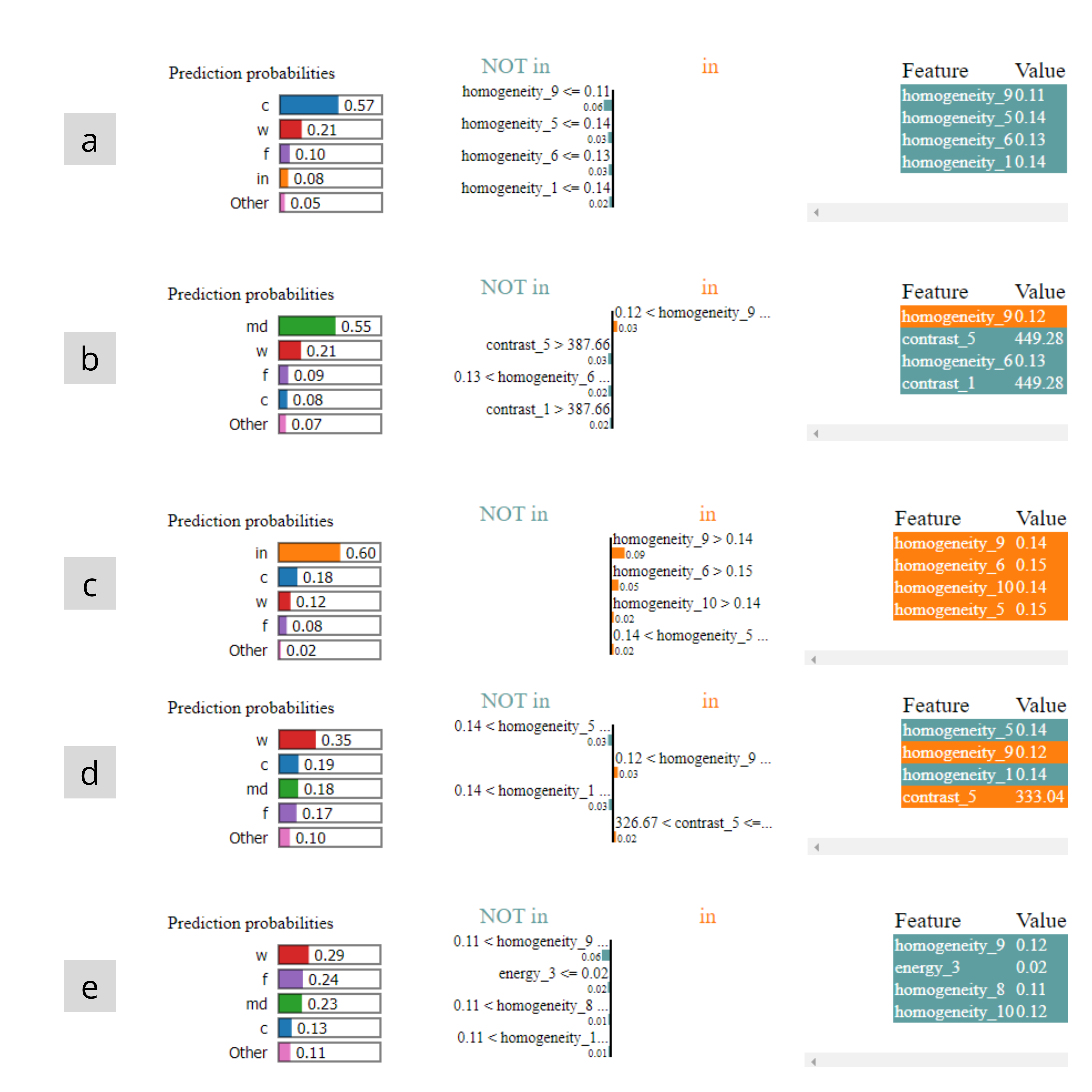

Supplement: Supplementary file 1 [file sensors-24-03198-s001.zip › FigureS5.png]

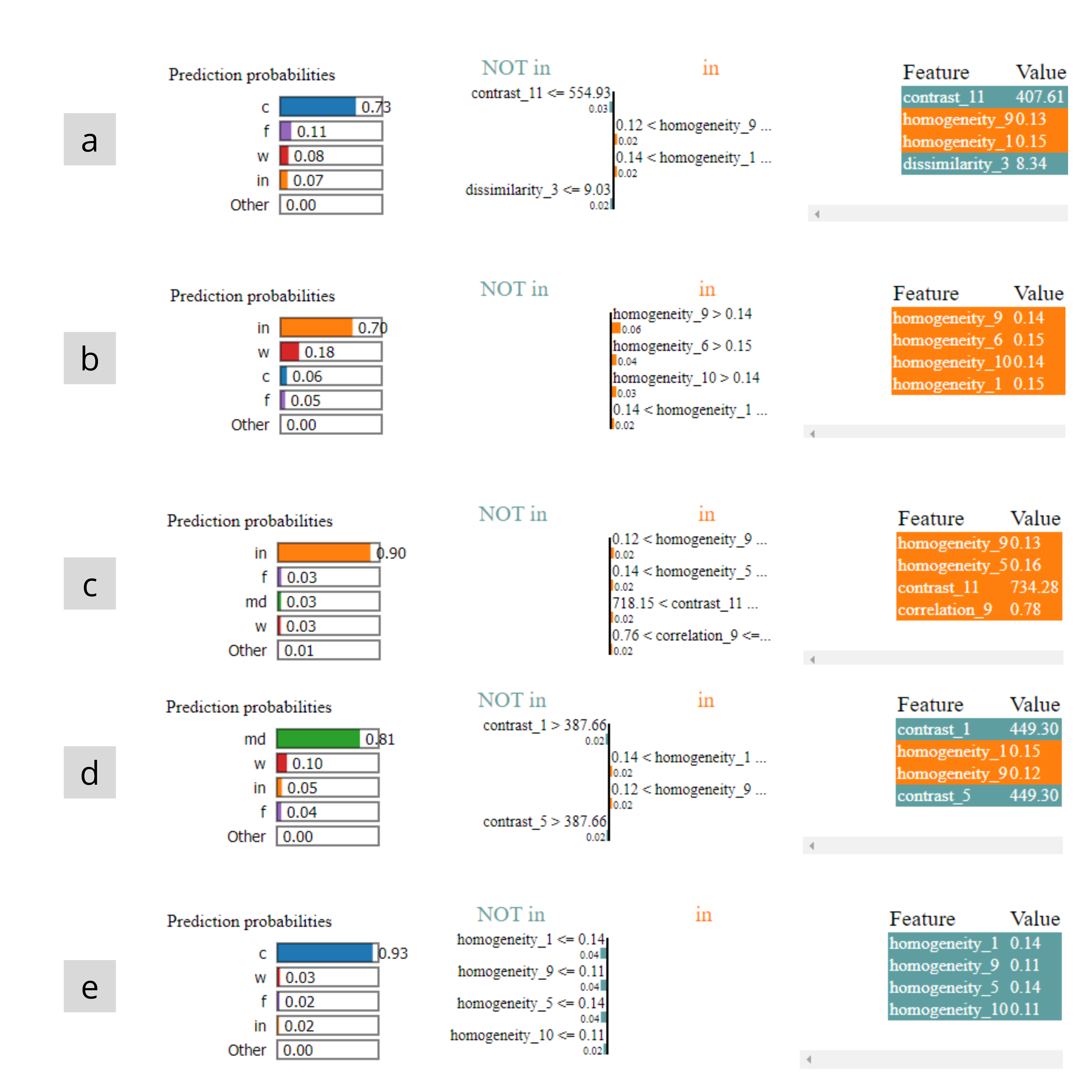

Supplement: Supplementary file 1 [file sensors-24-03198-s001.zip › FigureS6.png]

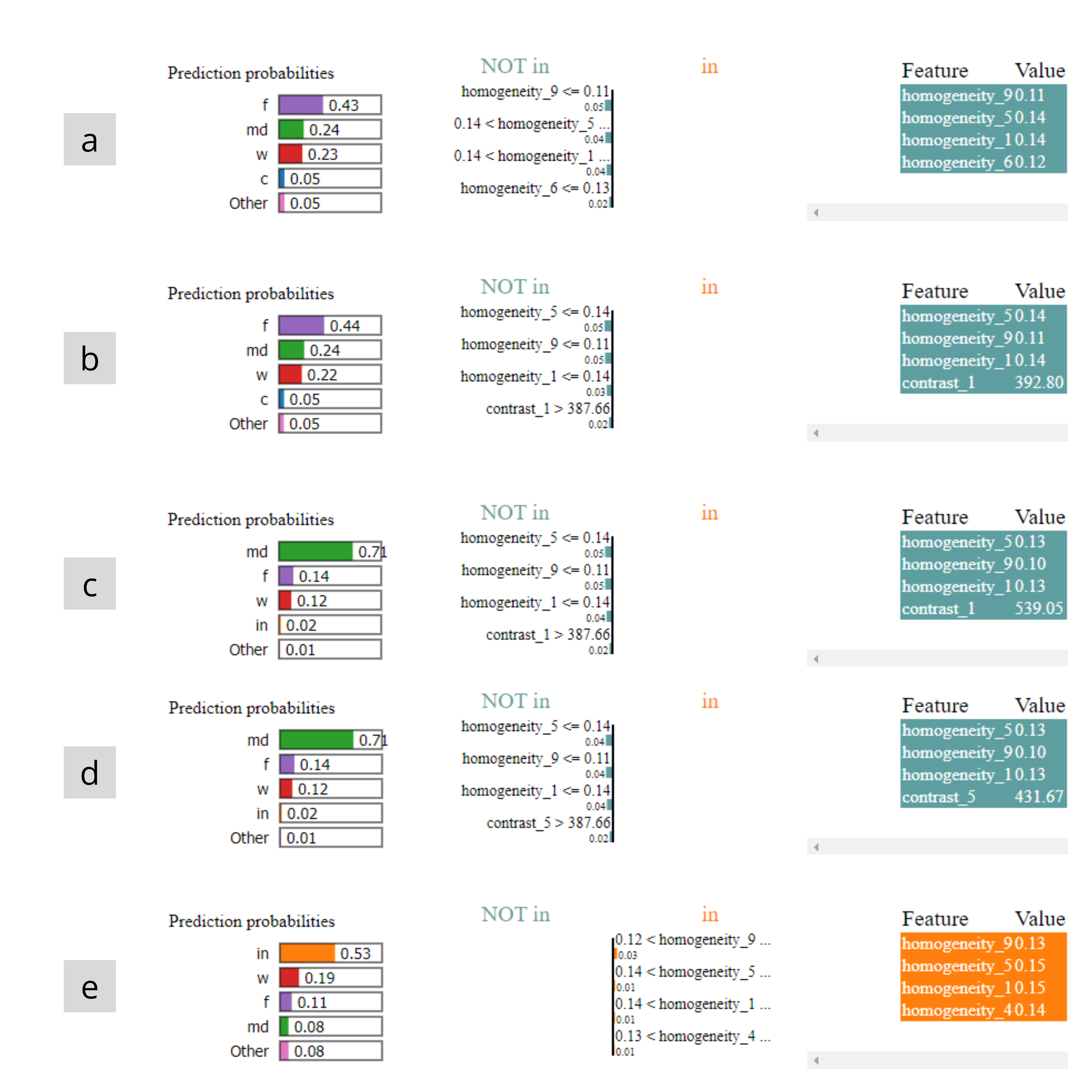

Supplement: Supplementary file 1 [file sensors-24-03198-s001.zip › FigureS7.png]

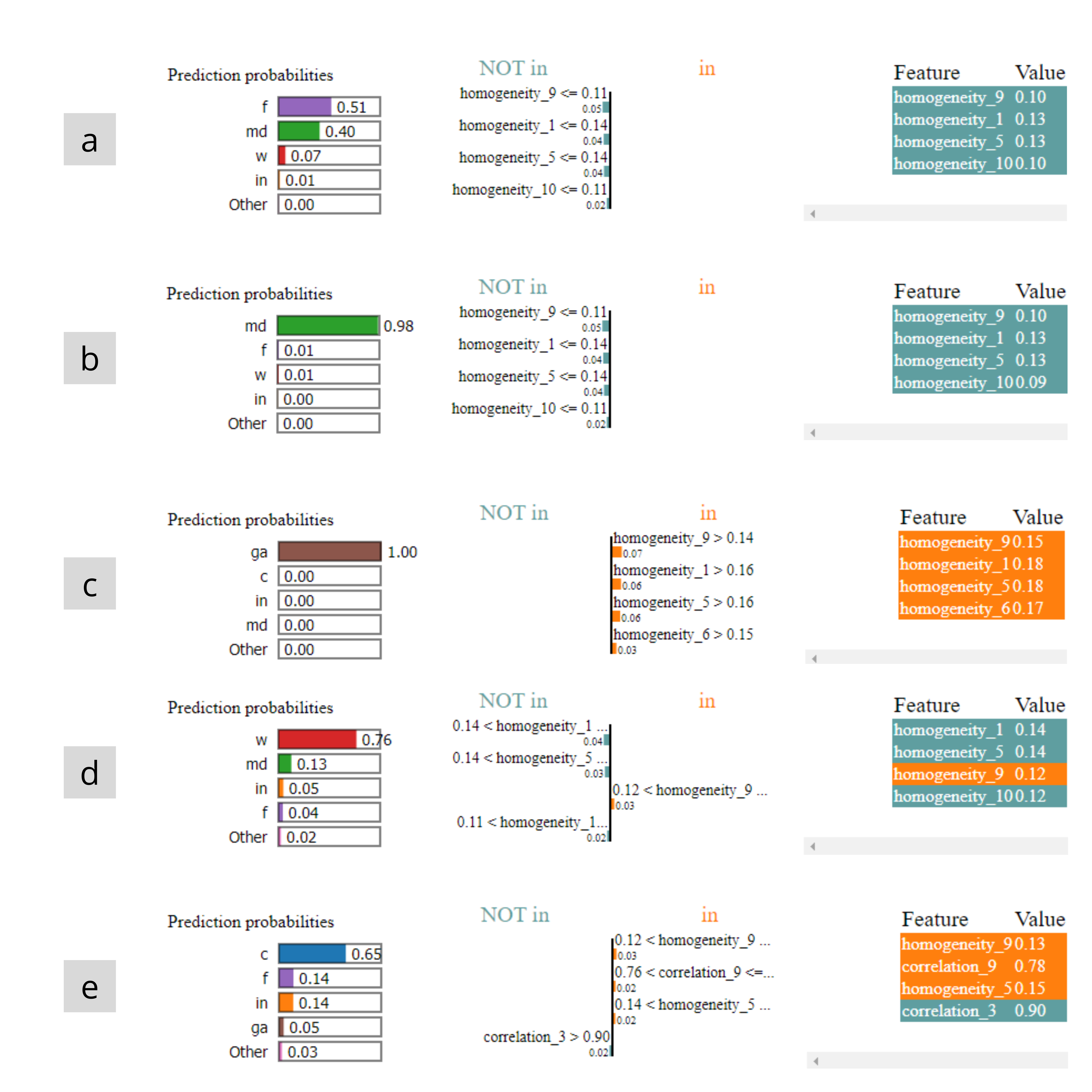

Supplement: Supplementary file 1 [file sensors-24-03198-s001.zip › FigureS8.png]

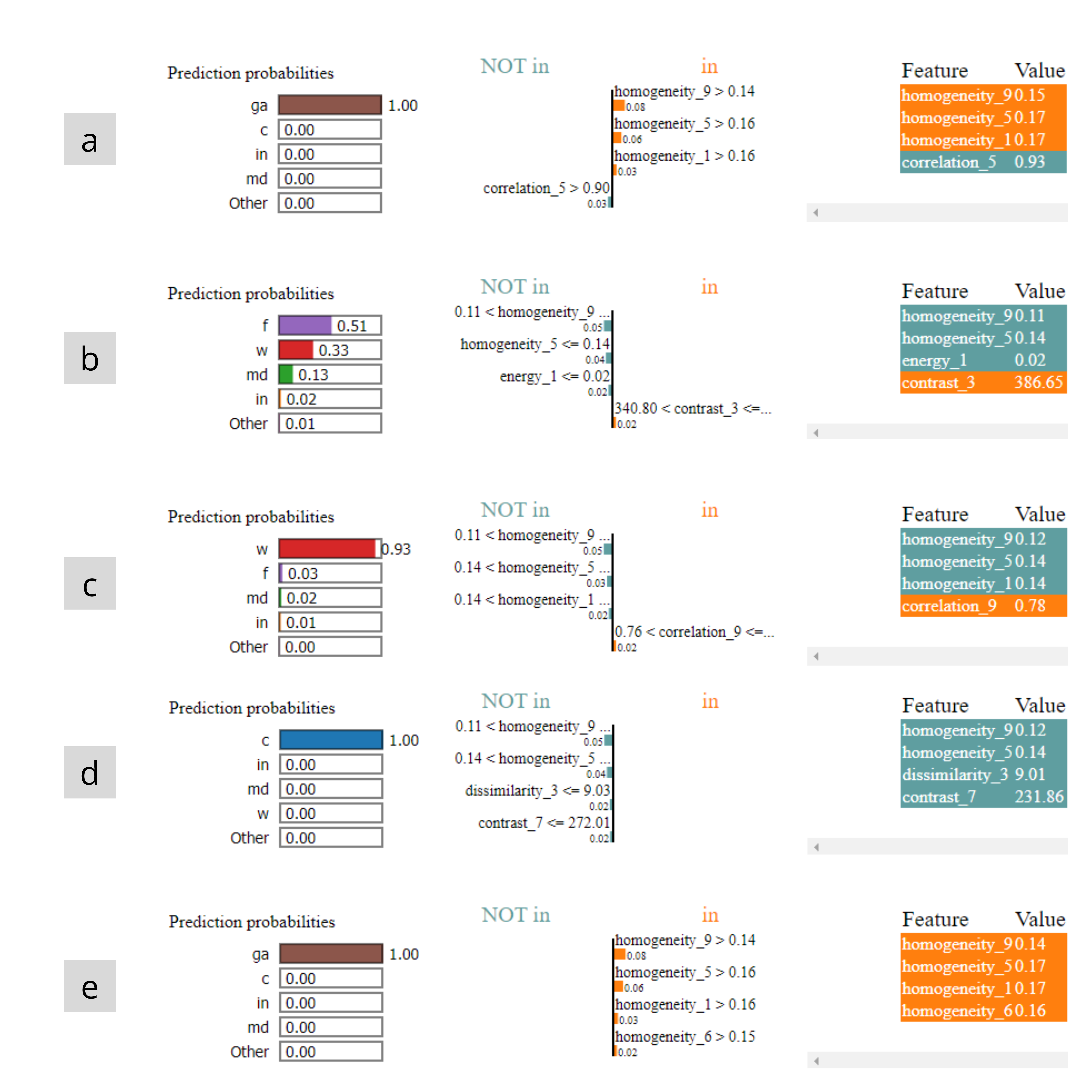

Supplement: Supplementary file 1 [file sensors-24-03198-s001.zip › FigureS9.png]
